# New Concepts for Vaccines^1^

**DOI:** 10.3201/eid1011.040797_06

**Published:** 2004-11

**Authors:** Trudy V. Murphy, Gary Dubin, Robert B. Belshe, Thomas P. Monath, Irene B. Glowinski, Susan A. Daniels, Joel C. Gaydos

**Affiliations:** * Centers for Disease Control and Prevention, Atlanta, Georgia, USA;; † GlaxoSmithKline, King of Prussia, Pennsylvania, USA;; ‡ St. Louis University Health Sciences Center, St. Louis, Missouri, USA;; § Acambis Inc., Cambridge, Massachusetts, USA;; ¶ National Institutes of Health, Bethesda, Maryland, USA;; # Department of Defense Global Emerging Infections Surveillance and Response System, Silver Spring, Maryland, USA

**Keywords:** vaccines, new vaccine approaches, platform technology

New technologies have created opportunities to develop novel vaccines, including vaccines for sexually transmitted infections, influenza, and arthropodborne viruses. A GlaxoSmithKline (GSK) vaccine for genital herpes combines a recombinant herpes simplex virus (HSV) glycoprotein D (gD) with AS04, a novel proprietary adjuvant containing aluminum salts, and 3-deacylated monophosphoryl lipid A. Two recently completed efficacy trials with the gD-AS04 vaccine demonstrated protection against infection with genital herpes in HSV-1 and -2 seronegative women, but not in seronegative men. Herpes simplex DNA and live attenuated or replication-impaired virus vaccines are in early development. Human papillomavirus (HPV) vaccine uses L1 virus-like particles (VLP) to immunize against HPV-16 and -18, two oncogenic types of HPV responsible for most cervical cancers. A HPV-16 L1 VLP vaccine (Merck Research Laboratories) and an HPV-16/18 L1 VLP vaccine (GSK) have recently been shown to prevent infection with the corresponding HPV types. In a double-blind, placebo-controlled efficacy trial, the GSK vaccine induced type-specific antibody responses that were higher than elicited by natural infections and resulted in protection against incident and persistent HPV-16 and -18 infections. Additional challenges for vaccines against sexually transmitted infections include assessing the duration of protection, identifying optimal target populations, and designing implementation strategies.

Cold-adapted, live attenuated strains of influenza have been developed in the United States (CAIV-T/USA) and Russia (CAIV-T/Russia) for use as vaccines. From master donor strains of influenza A or B, reassortant viruses can be produced annually with contemporary hemagglutinin (HA) and neuraminidase (NA) from wildtype influenza and six internal genes from the attenuated strains. Reactogenicity of the reassortants is generally mild; effectiveness is high. Expanded age indications are expected. Reverse genetics may be employed in the future to rapidly generate PR-8–based reassortant viruses. Reverse genetics could be used to eliminate the polybasic cleavage site of highly pathogenic avian influenza viruses and to rapidly make the PR-8 reassortants. New approaches to trivalent inactivated vaccine include high-dose HA vaccines, synthesis of recombinant HA antigen, addition of adjuvants, and intranasal transdermal administration and tissue culture production of whole viruses. Tissue culture production of antigen could shorten production time, increase the quantity of antigen, and exactly match the genetic sequence of HA in circulating viruses. Recombinant HA is effective as an immunogen and would reduce the reactogenicity associated with egg-grown trivalent inactivated vaccine. Use of adjuvants has the potential to stretch the vaccine supply in the event of vaccine shortage.

Platform technology makes use of live, recombinant viruses or bacteria to express one or more foreign genes that encode the antigen(s) of interest to deliver naked DNA encoding the vaccine antigen(s). The live vector may or may not be replication competent. Examples of such vector platforms include poxviruses, adenoviruses, alphavirus replicons, flaviviruses, and enteric bacteria (e.g., *Salmonella*, *Lactobacillus*). Preexisting immunity to the vector can limit the effectiveness of this approach, except with flaviviruses. In flavivirus vectors, e.g., yellow fever 17 D vaccine-virus platform, the genes encoding the antigens of the vector are entirely replaced by the genes of interest, e.g., West Nile virus, St. Louis encephalitis, Japanese encephalitis, or dengue virus. In clinical trials, several of these "chimeric" viral vaccines have been well-tolerated and highly immunogenic in clinical trials.

